# Glioma progression and recurrence involving maintenance and expansion strategies of glioma stem cells by organizing self-advantageous niche microenvironments

**DOI:** 10.1186/s41232-020-00142-7

**Published:** 2020-09-15

**Authors:** Tetsuya Taga, Kouichi Tabu

**Affiliations:** Department of Stem Cell Regulation, Medical Research Institute, Tokyo Medical and Dental University (TMDU) , 1-5-45, Yushima, Bunkyo-ku, Tokyo, 113-8510 Japan

**Keywords:** Autoschizis-like products, Cancer stem cell, Glioma, Glioma stem cell, Polymer, Stem cell niche, Tumor-associated macrophage

## Abstract

Due to the nature of enhanced resistance to conventional chemo/radiotherapies and metastasis, highly tumorigenic cancer stem cells (CSCs) have been proposed as a promising target for cancer eradication. To tackle the therapeutic difficulties of cancers involving CSCs, extensive research efforts have been directed toward understanding the extracellular microenvironments of CSCs, i.e., CSC niche, which plays important roles in CSC maintenance and expansion. Here we review recently identified mechanisms of maintenance and expansion of glioma CSCs (GSCs) leading to glioma progression and recurrence, with particular emphasis on the reports made by studies with a unique approach using polymer microarrays screening and with a unique viewpoint of necrotic particles. The polymer-based approach identified two groups of niche components, extracellular matrices (ECMs) and iron, and uncovered that co-expression of ECM-, iron-, and macrophage-related genes is predictive of glioma patients’ outcome. The study in view of a unique fraction of GSC-derived necrotic particles proposed that such particles develop GSC-supportive tumor-associated macrophages (TAMs). Taken together, these studies provide new insights into the mechanisms underlying GSC-driven niche development, i.e., organization of the self-advantageous niche microenvironments for GSC maintenance and expansion leading to glioma progression and recurrence. A series of such studies can redefine the current concept of anti-GSC niche therapy that targets ligands/receptors supporting GSCs, and have potential to accelerate cancer therapy development.

## Background

Malignant gliomas are the most frequent primary brain tumors in adults, and despite the progress of treatments including surgical resection and chemo/radiotherapies, the survival outcome has not been improved and far from desired [1]. According to the WHO criteria, glioblastoma, also known as glioblastoma multiforme or GBM, is the most malignant glioma classified as grade IV. Highly tumorigenic cancer stem cells (CSCs), due to their responsibilities for tumor progression, recurrence after conventional chemo/radiotherapies, and metastasis, have been proposed as a promising target for cancer eradication [2]. In order to tackle the therapeutic difficulties of cancers involving CSCs, much attention has focused on the extracellular microenvironments of CSCs, i.e., CSC niche, which is considered to maintain CSCs [3, 4]. Thus, disrupting the CSC niche is theoretically reasonable to impair the stem cell nature of CSCs and thereby inhibit the cancer recurrence. Under such circumstances, studies on the identification of key components of glioma CSC (GSC) niche have been made, but GSC niche remains to be fully elucidated due to its molecular and cellular complexity. Here we review GSC niche particularly by focusing on how GSCs organize their self-advantageous niche environments.

## Main text

### High-throughput polymer microarray screening to identify GSC niche components

From the current understanding of GSC niche, at least three anatomically distinct niches, i.e., perivascular, invasive, and perinecrotic niche, have been identified in GBM tissues representing functional heterogeneity of GSCs within a tumor [5]. Although researchers have thus studied a variety of GSC niche components, because of the remarkable molecular and cellular complexity, GSC niche does not seem to be fully defined. Recent advances in synthetic polymers have enabled us to mimic the microenvironments that afford normal stem cells the ability to be maintained with near physicochemical signals [6–8]. Synthetic polymers can function as biologically active scaffolds with controllable properties such as surface hardness, texture and roughness, and thus, for instance by using a thermo-modulatable technique, support long-term culture and serial passaging of human embryonic stem cells [9].

**Fig. 1:**
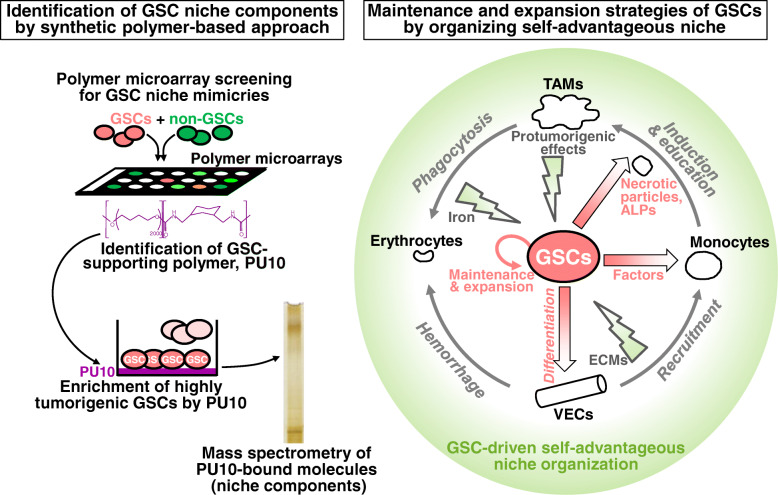
Self-maintenance strategies of GSCs by organization of the advantageous niche. GSC-driven niche development that leads to glioma progression and recurrence is illustrated based on the studies with unique polymer microarray screening and with a unique viewpoint of necrotic particles, ALPs. Left panel depicts how GSC niche components are identified by synthetic polymers: (1) SP-defined GSCs and non-GSCs containing red and green fluorescent protein genes were cultured on synthetic polymer microarrays. (2) The urethane-based polymer PU10 was found to support GSCs. (3) Highly tumorigenic GSCs among SP-defined GSCs were adhered to PU10. (4) PU-10-bound molecules were identified by mass spectrometry as candidate niche components. Right panel summarizes the GSC-driven self-advantageous niche organization, by combining polymer microarray screening outcomes and results of the study on a particular fraction of necrotic particles, ALPs. The polymer-based approach identified niche elements, ECMs and iron. ECMs that support GSCs are supplied by VECs differentiated from GSCs. Factors produced by GSCs efficiently direct host monocytes to iron storing and pro-tumoral macrophages. A particular fraction of necrotic products, ALPs, spontaneously arising from GSCs and non-GSC are engulfed by macrophages, among which those educated by ALPs from GSCs are suggested to function as protumoral TAM

Synthetic polymers have been applied to unveil GSCs and GSC niche, by screening acrylate-, acrylamide-, and urethane-based polymer scaffolds, which led to identification of a GSC niche mimicking polymer [10]. In this application, the Hoechst 33342 dye-effluxing side population (SP) cells, as GSCs, were isolated [10] from the rat glioma cell line C6 which had been characterized as a model for human GBM [11]. SP-defined GSCs and non-GSCs respectively expressing red and green fluorescent proteins (DsRed and GFP) were seeded and cultured on synthetic polymer microarrays containing some 376 polymers (Fig. 1, left top). Of the 173 polymer candidates that supported GSC attachment on day 4, the growth rates of GSCs and non-GSCs were evaluated and 5 top polymers with high GSC specificities were selected as hit polymers [10]. Among them, the urethane-based polymer PU10 synthesized from polytetramethylene glycol and 1,3-bis(isocyanatomethyl)cyclohaxane was found to support SP cells (SP-defined GSCs) most potently. It is of note that compared to PU10-non-adherent cells and the bulk SP-defined GSCs, PU10-adherent cells exhibited dramatically higher tumorigenic activity in NOD/SCID mice, suggesting that PU10 creates CSC niche environments, and also indicating that current conventional methods cannot fully distinguish the heterogeneity within the SP-defined GSCs (Fig. 1, left middle).

To identify niche elements that support highly tumorigenic PU10-adherent cells, serum- and/or cell-derived factors captured on PU10 were analyzed by mass spectrometry, and galectin-1 derived from rat C6 SP cells and bovine serum-derived transferrin (Tf) were identified (Fig. 1, left bottom) [10]. The former molecule, galectin-1 has been reported to facilitate the adhesion of glioma cells to extracellular matrices (ECMs) by cross-linking integrins, and its expression level correlates with glioma malignancies [12]. Importantly, galectin-1 is produced by SP cells, i.e., GSCs [10]. Concerning the latter molecule, the iron-carrier protein Tf, it is of note that the proliferation of SP cells, but not their descendants main population (MP) cells, is suggested to be regulated by Tf [10]. These results indicate that ECMs and Tf cooperatively maintain the SP pool and the tumor mass, and that polymer scaffolds are useful tools for predicting CSC niche elements. It is notable that C6 glioma SP cells, i.e., GSCs, express the higher level of Tf receptor than their progeny, MP cells (non-GSCs), and MP cells express the higher level of Tf and ECMs than SP cells.

### Self-maintenance and expansion strategies of glioma stem cells as revealed by polymer microarray screening

As mentioned above, from the polymer microarray screening and with the use of the hit polymer PU10, Tabu et al. revealed two groups of niche components, i.e., ECMs and iron [10], and reported that rat C6 GSCs are efficiently sustained in the presence of their differentiated progeny, MP cells (non-GSCs) expressing higher levels of ECMs and Tf. More important finding is that in xenografts of rat C6 GSCs, ECMs are supplied by the vascular endothelial cells (VECs), including those differentiated from transplanted GSCs, which have distinctively greater ability to retain a fluorescent antineoplastic agent mitoxantrone and resist to an anti-glioma drug temozolomide than host VECs [10]. In the rat C6 GSC xenografts, iron is stored in tumor-infiltrating host macrophages whose development is more efficiently facilitated by GSC-derived factors than non-GSC derived ones. Surprisingly, co-transplantation of rat C6 GSCs into the mouse brain with GSC factor-induced macrophages leads to the formation of tumors to a much greater extent than with non-GSC factor-induced macrophages. This indicates that GSCs, but not non-GSCs, have the exclusive potential to efficiently direct host monocytes to iron-storing and pro-tumoral macrophages.

The clinical significance of the niche elements identified by the study with polymer microarrays, i.e., ECMs, iron, and macrophages, is assessed by clinical database analysis, and it is clearly indicated that co-expression of ECM-, iron-, and macrophage-related genes is predictive of the outcome of patients with GBMs.

### Autoschizis-like product-mediated glioma recurrence, a new mode of self-organization of GSC niche

Spontaneous necrosis is a defining feature of GBM, and the extent of necrotic foci has been recognized as a highly reliable predictor for poor prognosis in GBM patients [13–15]. However, despite its strong correlations with poor prognosis, it remains still unclear whether necrosis could be a possible cause or mere consequence of glioma progression. Necrosis is morphologically characterized by membrane disruption, cytoplasmic swelling, organelle dysfunction, and karyolysis [16–19]. A number of studies have focused on the so-called immunogenic cell death of cancer cells induced by conventional chemo/radiotherapies, because dying cell-produced endogenous molecules known as damage-associated molecular patterns trigger anti-tumor Th1 response via dendritic cell engulfment [20–22].

A recent study has revealed the presence of a particular fraction of necrotic products spontaneously arising from glioma cells with severely disrupted plasma membrane and mesh-like cytoplasm, in which organelles mostly disappeared or were barely recognizable but in the form of small bodies [23]. Their nucleus has a spongiform structure and the salient margination of chromatin was observed along the nuclear envelope. These morphological alterations are broadly in accordance with those of a mode of cancer necrosis termed “autoschizis” which has a meaning of self-excision in Greek and was originally discovered as an induced cancer necrosis triggered by vitamin C and K3 treatments [24, 25]. Thus, the glioma necrosis partly includes autoschizis-like cell death and hence is named “autoschizis-like products (ALPs)” [23]. When administered to GM-CSF-primed bone marrow-derived macrophages/dendritic cells, ALPs were found to be specifically engulfed by macrophages expressing a tumor-associated macrophage (TAM) marker CD204. ALPs from GSCs had higher activity for the TAM development than those from non-GSCs. Of note, expression of the Il12b gene encoding a common subunit of interleukin (IL)-12/23 was upregulated in ALP-educated macrophages. Furthermore, IL-12 protein evidently enhanced the sphere-forming activity of GBM patient-derived cells, although interestingly, IL-12 is generally recognized as an antitumoral M1-macrophage marker. In silico analysis of transcriptome data of primary and recurrent GBMs revealed that higher expression of these IL-12 family genes was well-correlated with more infiltration of M1-type TAMs and closely associated with poorer prognosis in recurrent GBMs. These results highlight a role of necrosis in GSC-driven self-beneficial niche construction and glioma progression, providing important clues for developing new therapeutic strategies against gliomas.

Tumor cell death during tumor progression by, for instance, mechanical, physiological, chemical, and tumor microenvironmental stress may apparently be disadvantageous for the tumor, but this study revealed the self-expanding strategies of GSCs by constructing GSC-supporting niche via necrotic cell death products called “ALPs.” Concerning apoptosis, a recent study has reconciled the paradox that a higher number of apoptotic GBM cells positively correlates with decreased patients’ survival; apoptotic GBM cells secrete extracellular vesicles including oncogenic spliceosomes toward surviving GBM cells [26].

Again, in conclusion, autoschizis is a type of cell death which is characterized by a decrease in cell size, cytoplasmic self-excisions, and nuclear and nucleolar morphologic degradations without the formation of apoptotic bodies, induced by ascorbic acid or menadione (vitamin C or vitamin K3) [24, 25, 27]. To the best of our knowledge, autoschizis has not been reported to be induced by conventional antineoplastic agents in the literature to date. Reference [23] cited in the manuscript is the first paper about the presence of spontaneous autoschizis-like cell death, in which “-like” is appended to “autoschizis” because of the absence of vitamin C/vitamin K3. Cytotoxicity of vitamin C has been explained by superoxide radical (O_2_–) generated by a mechanism involving vitamin C [28]. Concerning the mechanism governing necrosis, superoxide dismutase and Fenton reaction transform O_2_– into hydrogen peroxide (H_2_O_2_) and extremely reactive hydroxyl radical (·OH), so-called reactive oxygen species (ROS) [29]. On the other hand, the mechanisms underlying the formation of necrotic foci in GBMs as a spontaneous cell death induced by vascular occlusion (thrombosis) and subsequent lack of oxygen supply were reported [15, 30]. Considering that hypoxia triggers O_2_– production via mitochondrial complex IV and stabilizes HIFs [31], it would be more reasonable to conclude that glioma necrosis is induced by hypoxia-driven iron-dependent oxidative stress with no exposure to vitamin C [24, 25, 27]. It is also important to note that enhanced resistance of GSCs/CSCs to chemo/radiotherapies is now widely recognized [1, 3]. ALPs, if any, were to be emerged from bulk glioma cells treated by chemo/radiotherapies might be those from non-GSCs, which exhibit much lower activity for the TAM development than those from GSCs [23]. Thus, this study revealed the self-expanding strategies of GSCs by constructing GSC-supporting niche via a particular fraction of products of spontaneous necrotic cell death, called “ALPs” from GSCs [23].

Further elucidation of functional heterogeneity of GSCs, including how GSCs adopt the different fates, dying supporter, and surviving driver, will open a new therapeutic window for glioma eradication [23].

## Conclusion

This article has reviewed mechanisms of maintenance and expansion of glioma CSCs (GSCs) revealed recently, which lead to glioma progression and recurrence, by focusing on studies with unique polymer microarrays screening and with a unique viewpoint of necrotic particles, ALPs. The demonstration of the utility of synthetic polymer scaffolds as a tool for cancer (stem cell) research has provided important insights into therapeutic strategies targeting CSC niche by proposing that co-expression of ECM-, iron-, and macrophage-related genes is predictive of glioma patients’ outcome. Thus, such an approach with synthetic polymers will contribute to the refinement of the GSC maintenance/expansion concept. In addition, the study on a fraction of GSC-derived necrotic particles designated as ALPs has revealed that ALPs function as a key mediator of the development of a GSC-supportive M1-type TAMs. This work thus demonstrates that glioma necrosis is not a meaningless death but is a tumor-beneficial event. Taken together, the studies covered by this review provide new insights into the mechanisms underlying GSC-driven niche development as well as glioma progression and recurrence (Fig. 1, right).

## Data Availability

Further information and requests for resources and reagents should be directed to the authors: Tetsuya Taga (taga.scr@mri.tmd.ac.jp) and Kouichi Tabu (k-tabu.scr@mri.tmd.ac.jp).

## Abbreviations

ALP: Autoschizis-like product
CSC: Cancer stem cell
ECM: Extracellular matrix
GBM: Glioblastoma multiforme
GSC: Glioma stem cell
IL-12: Interleukin-12
MP: Main population
SP: Side population
TAM: Tumor-associated macrophage
Tf: Transferrin
VEC: Vascular endothelial cell
